# Evolution, ecology and systematics of *Soldanella* (Primulaceae) in the southern Apennines (Italy)

**DOI:** 10.1186/s12862-015-0433-y

**Published:** 2015-08-11

**Authors:** Alessandro Bellino, Leonardo Bellino, Daniela Baldantoni, Antonio Saracino

**Affiliations:** Dipartimento di Chimica e Biologia, Università degli Studi di Salerno, Via Giovanni Paolo II, 132, Fisciano, 84084 Salerno Italy; Dipartimento di Agraria, Università degli Studi di Napoli “Federico II”, Via Università, 100, Portici, 80055 Naples Italy

## Abstract

**Background:**

The populations of *Soldanella* (Primulaceae) of the southern Apennines (Italy) are unique within the genus for their distribution and ecology. Their highly fragmented distribution range, with three main metapopulations on some of the highest mountains (Gelbison, Sila and Aspromonte massifs) of the area, poses intriguing questions about their evolutionary history and biogeography, and about the possibility of local endemisms.

**Aims and methods:**

In order to clarify the phylogeny and biogeography of the three metapopulations of *Soldanella* in the southern Apennines, attributed to *S. calabrella* to date, and to identify possible local endemisms, a comparative approach based on the study of molecular, morphological and ecological characteristics of the populations was employed. Specifically, one nuclear (total ITS) and two plastid (*rbcL* and *trnL*) markers were used for the phylogenetic analyses, performed through both maximum likelihood and Bayesian techniques. Among the morphological features, the glandular hair and leaf biometric traits were analysed, and the environment in which the populations grew was characterised for altitude, forest canopy composition and soil pH, C, N and organic matter.

**Results and conclusions:**

Our findings demonstrate that the lineage of *Soldanella* of southern Italy diverged from the Carpathians lineage during the Middle Pleistocene, and underwent an evolutionary radiation during the Late Pleistocene. The populations of the Sila and Aspromonte massifs diverged from the populations of the Gelbison massif around 380000 years ago and are probably undergoing a progressive differentiation due to their isolation. The populations on the Gelbison massif, moreover, have different morphological features from those of the Sila and Aspromonte massifs and a different ecological niche. The molecular, morphological and ecological data clearly demonstrate that the metapopulation of *Soldanella* on the Gelbison massif belongs to a new taxonomic unit at the species level, which we name *Soldanella sacra* A. & L. Bellino from the name of the massif on which it was discovered, the “Holy Mountain”.

**Electronic supplementary material:**

The online version of this article (doi:10.1186/s12862-015-0433-y) contains supplementary material, which is available to authorized users.

## Background

*Soldanella* is a small genus of European orophytes belonging to the Primulaceae family. The genus takes its name from the round and cordate shape of the leaves, that resemble a small coin (from the Latin *soldo* = “coin” [1]) and includes, according to the most recent taxonomic and nomenclature revisions [2, 3], 16 species and 4 subspecies. Later, however, Niederle [4] described a new species from the Tatra Mts, *S. tatricola*. The distribution range of the genus covers all the major European mountain chains but most of the species of *Soldanella* are endemic to very limited areas, often comprising a single mountain chain or massif [5]. From an evolutionary point of view, *Soldanella* forms a monophyletic clade with the genera *Hottonia* and *Omphalogramma* [6, 7] and, according to Zhang and co-workers [5], underwent an evolutionary radiation during the Quaternary, after the ancestor of the genus, probably close to *S. villosa* Darracq., came to Europe from the East.

Although the genus is well circumscribed within the Primulaceae in relation to the floral traits, the species delimitation is often uncertain, due to the remarkable homogeneity of the morphology and the ecology of many species. On a morphological basis, the genus was subdivided into two sections, namely sect. *Soldanella* Pawlowska (ex. sect. *Crateriflores* (Borbás) Knuth) and sect. *Tubiflores* (Borbás) Knuth, while on an ecological basis it is generally classified into “montane” and “alpine” species, roughly matching the morphological subdivision [2, 3]. Recently however, the paraphyly of sect. *Soldanella* with respect to a polyphyletic sect. *Tubiflores* and the independent evolution of floral reduction in the species of the latter were demonstrated [5, 8].

According to the extensive revision of the genus by Zhang and Kadereit [2], four species of *Soldanella* are found in Italy, one of which with two subspecies: *S. alpina* ssp. *alpina* L., *S. calabrella* Kress, *S. pusilla* ssp. *pusilla* Baumg., *S. minima* ssp. *minima* Hoppe and *S. mimima* ssp. *samnitica* Cristofolini et Pignatti. The distribution of the Italian species is widely discontinuous. With the exception of *S. calabrella*, these species are mainly distributed over the Alps and some of the highest mountains of the northern and central Apennines. Conversely, *S. calabrella* is the unique species present in the southern Apennines, with two main distribution areas on the Sila and Aspromonte massifs (Calabrian Apennines), and appears to be phylogenetically close to the species of the Carpathians based on ITS sequencing [2, 5].

Recently, a population of *Soldanella* sp. was discovered on the Gelbison massif, in the Campanian Apennines, in southern Italy, and attributed to *S. calabrella* by Azzella and Burrascano [9]. The attribution of the population on the Gelbison massif to *S. calabrella*, was based on the observation of the glandular hairs, that are considered the main differential trait among the species of *Soldanella* [2]. This report extends the distribution range of the species, which can no longer be considered to be endemic of the Calabrian Mts.

The geographical isolation among the populations of *Soldanella* in southern Italy, however, poses intriguing questions about their phylogenetic relationships and about the possibility of vicariance processes. Moreover, the Gelbison massif has different geological characteristics [10] and vegetation types [11] as compared to the Calabrian mountains, which could have favoured the differentiation of local endemisms. Based on these considerations, we hypothesise that the populations of the Gelbison massif and those of the Sila and Aspromonte massifs could constitute different evolutionary and taxonomic units. As such, we will refer to the populations analysed generically as *Soldanella* sp.

With the aim to clarify the evolution of the populations of *Soldanella* sp. from southern Italy, identify possible local endemisms and provide a detailed analysis of their ecology and systematics, we studied 15 populations from the three main distribution areas of the genus in southern Italy, using a comparative approach based on molecular, morphometric and ecological traits. Due to the availability of the ITS sequences for all the species of the genus [5, 8] with the exception of *S. tatricola* Niederle, we thoroughly revised them in a phylogeographical framework, in order to draw a scenario for the evolutionary history of *Soldanella* in Europe, with a special focus on the early diversification events and on the evolution of the southern Apennines populations. Moreover, despite the numerous potential threats to the persistence of the southern Apennines *Soldanella* sp. populations, related to the restricted areas to which they belong, no official assessment of their conservation status has been provided yet, not even for the widely known *S. calabrella* populations of the Calabrian Apennines. To partially fill this gap, we provided an estimation of the conservation status and priorities for the recently discovered metapopulation of the Gelbison massif, suggesting possible strategies for its management.

## Results

### Ecology

**Fig. 1:**
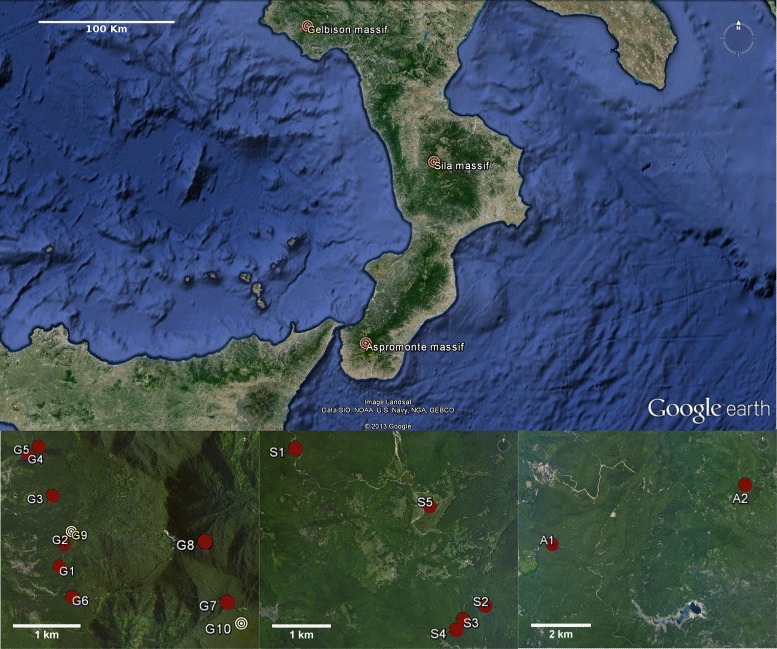
Studied populations. Satellite image of the southern Apennines, showing the three massifs from which the populations of *Soldanella* sp. were studied. The three subfigures show the distribution of the sampled populations on the Gelbison (*left*), the Sila (*center*) and the Aspromonte (*right*) massifs. Populations marked in yellow on the Gelbison massif were not included in the present study

All the populations of *Soldanella* analysed (Fig. 1) were associated to wetlands, differing in their characteristics across the distribution range. The habitat of the populations on the Gelbison massif is represented by resurgences and related perennial brooks from 840 to 1400 m a.s.l. (Table 1), where plants mainly colonise the brook bed and its sides within a range of ∼30 cm from water. The brook sectors colonised flow under open canopies of *Alnus cordata* Loisel., *Alnus glutinosa* L., *Castanea sativa* Miller and sometimes *Fagus sylvatica* L. (Table 1). Conversely, the populations on the Sila massif mainly colonised water meadows, always under *F. sylvatica* and *Abies alba* Miller canopies or in open areas, from 1670 to 1780 m a.s.l. (Table 1). Similarly, the populations on the Aspromonte massif colonised water meadows or stream sides, mainly on steep slopes and within a range of approximately 4 m from water. The populations were distributed under continuous and discontinuous *F. sylvatica* and *A. alba* canopies, from 1400 to 1835 m a.s.l. (Table 1).

**Table 1:** Geographical location, altitude and tree canopy composition of the 15 studied populations of *Soldanella* sp.

		Latitude	Longitude	Altitude	Stand composition
		(Degree North)	(Degree East)	(m a.s.l.)	
Gelbison	G1	40°12’ 49.7"	15°18’ 55.7"	900	C, Ac, Ag
	G2	40°12’ 49.7"	15°18’ 58.5"	940	C, Ac
	G3	40°13’ 23.5"	15°18’ 50.5"	945	C, Ac, Ag
	G4	40°13’ 46.0"	15°18’ 33.3"	840	C, Ac, Ag
	G5	40°13’ 50.1"	15°18’ 41.0"	866	C, Ac, Ag
	G6	40°12’ 32.2"	15°19’ 0.6"	1130	Ac, Ag
	G7	40°12’ 31.4"	15°20’ 43.7"	1315	F
	G8	40°13’ 0.0"	15°20’ 28.6"	1400	F
Sila	S1	39°18’ 55.7"	16°24’ 11.6"	1720	F, A
	S2	39°17’ 28.3"	16°26’ 28.0"	1700	F
	S3	39°17’ 21.7"	16°26’ 11.0"	1760	F
	S4	39°17’ 16.0"	16°26’ 5.6"	1780	F, A
	S5	39°18’ 23.9"	16°25’ 48.4"	1670	O
Aspromonte	A1	38°8’ 38.0"	15°50’ 27.2"	1400	F, A
	A2	38°9’ 30.8"	15°54’ 52.1"	1835	F, A

*A* *Abies alba*, *Ac* *Alnus cordata*, *Ag* *Alnus glutinosa*, *C* *Castanea sativa*, *F* *Fagus sylvatica*, *O* water meadow without canopy cover

**Fig. 2:**
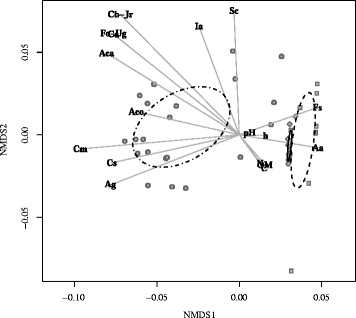
Ecological differentiation among the studied populations. Non-metric Multidimensional Scaling based on the ecological data. Circles, squares and diamonds represent the observations belonging to the Gelbison, the Sila and the Aspromonte massifs, respectively. Confidence ellipses (for *α* = 0.05) for the three provenances (dotted-dashed: Gelbison; dashed: Sila; solid: Aspromonte) are also shown. Aa: *Abies alba*, Aca: *Acer cappadocicum*, Aco: *Alnus cordata*, Ag: *Alnus glutinosa*, Ca: *Corylus avellana*, Cb: *Carpinus betulus*, Cm: *Crataegus monogyna*, Cs: *Castanea sativa*, Fo: *Fraxinus ornus*, Fs: *Fagus sylvatica*, Ia: *Ilex aquifolium*, Jr: *Juglans regia*, Sc: *Salix caprea*, Ug: *Ulmus glabra*, C: soil carbon, N: soil nitrogen, OM: soil organic matter, pH: soil pH, h: site elevation

NMDS analysis based on the ecological data highlighted a clear cut differentiation between the metapopulation of the Gelbison massif and those of the Sila and Aspromonte massifs, of which confidence ellipses adjoin each other (Fig. 2). All variables, particularly *A. cordata*, *A. alba*, soil pH and site elevation, contribute to the differentiation of the populations from the three provenances. In relation to the soil rhizosphere characteristics and site elevation, the populations on the Gelbison massif colonised soils at lower (*P*<0.001) altitude, with higher (*P*<0.001) pH and lower (*P*<0.05) C and organic matter than the populations on the Sila and Aspromonte massifs, of which soils did not significantly differ for any parameter analysed.

The phenology also differed among the populations, with a shift in the flowering period of about 1 month. The flowering period of the populations of *Soldanella* on the Gelbison massif extended from April to May (preceding foliation of canopy of the broad-leaved trees by about 1 month) whereas the populations on the Sila and Aspromonte massifs flowered from the end of May to July. Seed dispersal took place from June to July on the Gelbison massif and approximately 1 month later in the Calabrian Apennines.

### Molecular analyses

The *rbcL*-amplified region of *Soldanella* sp. was 1267 bp long in all the samples. Two haplotypes were observed among the 45 sequences analysed. The populations from the Sila and the Aspromonte massifs showed the same sequence, whereas the populations from the Gelbison massif showed a transversion A <— > C at alignment position 960. The same transversion with respect to the populations of the Calabrian Apennines was shared by *S. pusilla*. For *trnL*, two haplotypes were observed among the 21 sequences analysed, with the same geographical distribution described for *rbcL*. The *trnL*-amplified region was 469 bp long for the haplotype of the Gelbison massif and 470 bp long for the haplotype of the Sila and Aspromonte massifs, due to the presence of a poly-A microsatellite being 10 bp long in the former and 11 bp long in the latters. In addition, the haplotype from the Gelbison massif showed a transition A <— > G at alignment position 427, and differentiated from the *trnL* sequence of *S. pusilla* uniquely by the length of the poly-A microsatellite, being 9 bp long in *S. pusilla*. Total ITS sequences were always 890 bp long and showed 4 different ribotypes, 1 for the populations of the Gelbison massif, 2 for the populations of the Sila massif and 1 for the populations of the Aspromonte massif. The ribotypes of the Gelbison and the Sila massifs showed a transition A <— > T at alignment position 149 with respect to the ribotype of the Aspromonte massif. The samples from the Sila massif, in addition, belonged to two ribotypes, one identical to the ribotype of the Gelbison massif and the other showing a transversion G <— > T at alignment position 240.

**Fig. 3:**
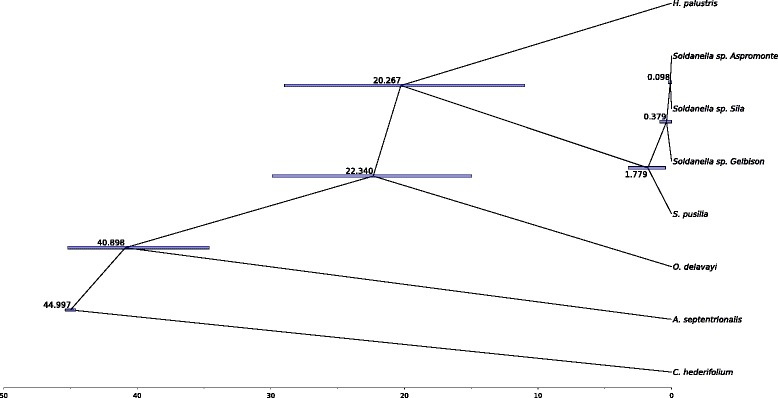
Multispecies coalescent tree. Maximum credibility tree from the multispecies coalescent model based on the three markers analysed. The mean age estimates for each node are reported along with their 95 % HPD intervals as node bars. All the nodes have 100 % posterior support, with the exception of the clade comprising *Soldanella sp. Sila* and *Soldanella sp. Aspromonte*, that has 83.5 % posterior support. Axis scale is in million of years

The multispecies coalescent model placed the haplotype from the Gelbison massif as the sister group of a cluster comprising the haplotypes from the Sila and Aspromonte massifs (Fig. 3). The mean age estimate of the *Soldanella* lineage was 20.21 My, 95 % HPD: 11.12 - 29.00 My, whereas for the evolutionary radiation of the genus it was 1.78 My, 95 % HPD: 0.46 - 3.24 My. The differentiation of the haplotype of the Gelbison massif from those of the Sila and Aspromonte massifs was dated to 0.38 My ago, 95 % HPD: 0.01 - 0.88 My (Fig. 3).

**Fig. 4:**
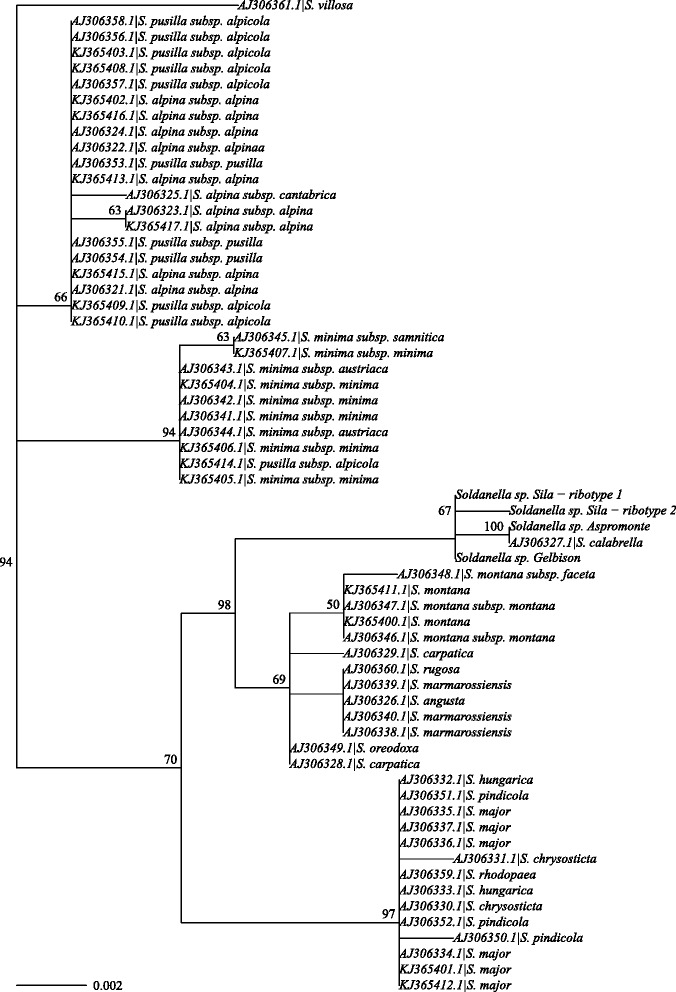
ITS maximum likelihood tree. Maximum likelihood tree for the ITS data. Node labels indicate bootstrap support (shown only for values >50 %), scale bar indicates substitutions per site

**Fig. 5:**
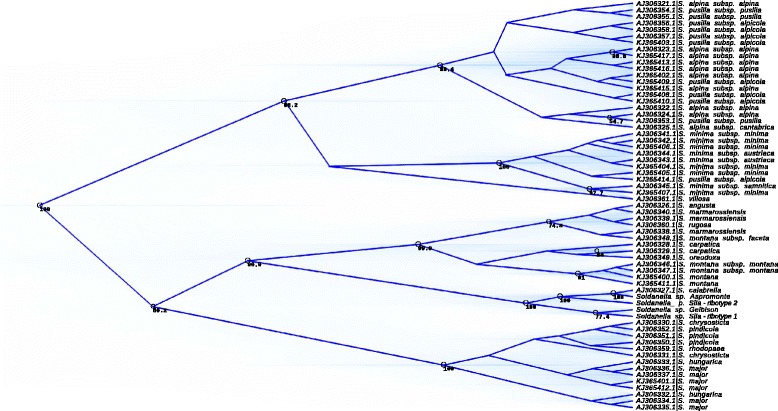
DensiTree plot for the ITS data. DensiTree plot for the calibrated Bayesian tree based on the ITS data. The consensus trees are drawn with the superimposition of the root canal. Node labels indicate posterior support (shown only for values >50 %). Axis scale is in million of years

**Fig. 6:**
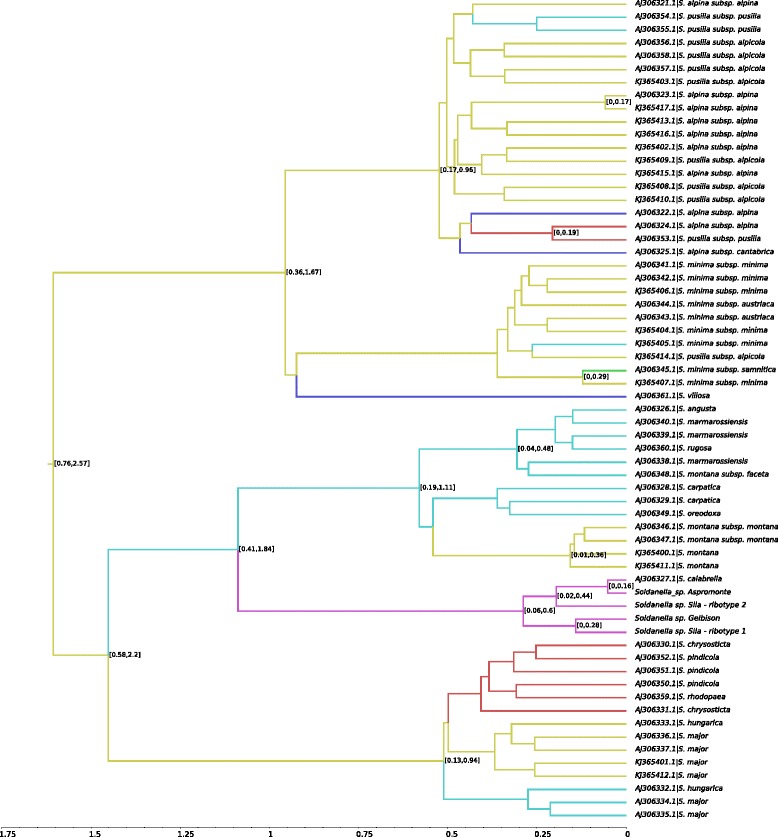
Soldanella evolution during the Pleistocene. Bayesian Maximum Credibility Tree for the ITS data. Branches are colored according to their most probable provenance (central Europe/Alps: yellow; Carpathians: cyan; Balkans: red; Pyrenees: blue; central Italy: green; southern Italy: purple). The 95 % HPD on node ages is reported only for nodes with posterior support >50 % (for node support please refer to Fig. 5). Axis scale is in million of years

ITS failed to differentiate among numerous species of *Soldanella* (Figs. 4 and 5). Overall, four main lineages were resolved: one containing the species of the southern Apennines and the others corresponding roughly to species of the i) Carpathians, ii) Balkans and iii) central Europe, Pyrenees, Alps and northern and middle Apennines (Fig. 6). The clades were poorly resolved at the species level, particularly those comprising the species of the Balkans and of the Alps and central Europe. The most notable exception is the clade comprising the species of *Soldanella* from the southern Apennines, which had support >75 % for all the internal nodes (Figs. 5 and 6). In particular, the ribotypes of the three metapopulations analysed were grouped into two sister groups, one comprising the ribotype of the Gelbison massif and one of the two of the Sila massif, and another group comprising the ribotypes of the Aspromonte massif and the other one of the Sila massif.

The phylogeographical analysis based on the ITS data indicated central Europe (42.1 %) as the most probable root provenance of the *Soldanella* genus, although the Carpathians (30.5 %) had comparable support (Additional file 1: Video S1). Among the other provenances, the Balkans had a posterior probability (14.4 %) similar to the prior probability, whereas each of the other provenances had support <0.1 %. The populations of southern Italy diversified 1.09 My (95 % HPD: 0.41–1.84 My) from the lineage of the Carpathians (45.4 % support) (Fig. 6), although a European origin cannot be ruled out (29.7 % support). The Apennines were colonised in at least two different events: an early one reaching the southern Apennines, and a recent one from central Europe-Alps involving the colonisation of the central Apennines by *S. minima* ssp. *samnitica* (Additional file 1: Video S1).

### Morphometry

**Fig. 7:**
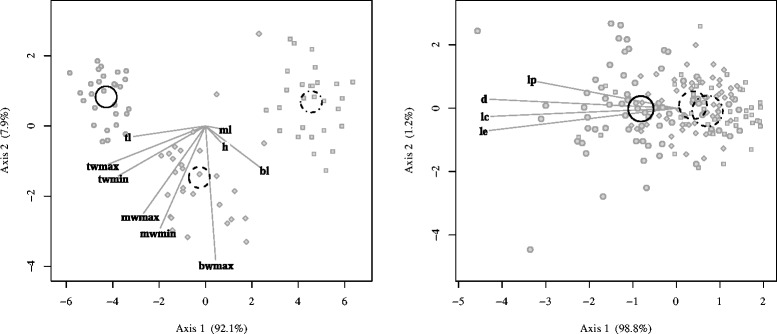
Morphometric differentiation of the studied populations. Canonical Variates Analysis of the glandular hair (left) and leaf (right) biometrical traits. Circles, squares and diamonds represent the observations belonging to the Gelbison, the Sila and the Aspromonte massifs, respectively. Confidence circles (for *α* = 0.05) for the three provenances (*dotted-dashed*: Gelbison; *dashed*: Sila; *solid*: Aspromonte) are also shown. For the abbreviations, refer to the Methods section

The populations from the three provenances were clearly differentiated based on the biometrical traits of the glandular hairs. In particular, the glandular hairs from the populations of the Gelbison massif were shorter than those from the populations of the Calabrian Apennines, had longer and wider terminal cells as well as shorter basal and middle cells (Table 2, Fig. 7). The biometrical traits of the terminal cell differentiated also the populations of the Aspromonte massif from those of the Sila massif, with values in between the other two.

**Table 2:** Mean ± SE of each environmental variable and biometrical trait analysed in the populations of the Gelbison, the Sila and the Aspromonte massifs

		Gelbison	Sila	Aspromonte
Soil		*n* = 23	*n* = 15	*n* = 11
	pH	6.96 ± 0.11^*a*^	5.67 ± 0.11^*b*^	5.98 ± 0.10^*b*^
	OM (% d.w.)	15.16 ± 2.49^*a*^	31.65 ± 6.45^*b*^	31.73 ± 3.77^*b*^
	Carbon (% d.w.)	7.54 ± 1.35^*a*^	14.02 ± 3.33^*b*^	15.31 ± 1.82^*b*^
	Nitrogen (% d.w.)	0.62 ± 0.10^*a*^	0.93 ± 0.22^*a*^	1.01 ± 0.11^*a*^
Glandular hairs		*n* = 28	*n* = 28	*n* = 29
	h (*μ*m)	102.69 ± 2.83^*a*^	109.76 ± 2.38^*a*^	109.02 ± 3.73^*a*^
	tl (*μ*m)	41.00 ± 0.93^*a*^	29.15 ± 0.94^*b*^	36.37 ± 1.09^*c*^
	twmax (*μ*m)	35.05 ± 0.33^*a*^	22.37 ± 0.36^*b*^	31.46 ± 0.57^*c*^
	twmin (*μ*m)	18.92 ± 0.25^*a*^	13.75 ± 0.20^*b*^	17.87 ± 0.34^*c*^
	ml (*μ*m)	31.68 ± 0.98^*a*^	34.14 ± 0.96^*a*^	32.99 ± 1.25^*a*^
	mwmax (*μ*m)	21.39 ± 0.32^*a*^	17.55 ± 0.31^*b*^	22.07 ± 0.49^*ac*^
	mwmin (*μ*m)	18.53 ± 0.35^*a*^	16.17 ± 0.22^*b*^	19.91 ± 0.47^*c*^
	bl (*μ*m)	28.13 ± 1.03^*a*^	37.49 ± 1.17^*b*^	35.38 ± 1.24^*b*^
	bwmax (*μ*m)	20.52 ± 0.47^*a*^	21.59 ± 0.37^*ab*^	25.50 ± 0.67^*b*^
Leaves		*n* = 69	*n* = 48	*n* = 58
	le (mm)	2.19 ± 0.09^*a*^	1.38 ± 0.09^*b*^	1.53 ± 0.05^*b*^
	lc (mm)	2.98 ± 0.11^*a*^	1.89 ± 0.12^*b*^	2.11 ± 0.09^*b*^
	d (mm)	3.49 ± 0.13^*a*^	2.24 ± 0.14^*b*^	2.52 ± 0.08^*b*^
	lp (mm)	8.68 ± 0.91^*a*^	3.85 ± 0.31^*b*^	5.06 ± 0.20^*b*^

Different letters indicate significant differences (for *α* = 0.05) according to the Tukey HSD *post hoc* test. The number of observations for each group of data (*n*) is also reported. For the abbreviations, refer to the Methods section

The leaf traits also differentiated the populations of the Gelbison massif from those of the Calabrian Apennines. Specifically, the populations from the Gelbison massif had the longest petioles and the greatest leaf surface, whereas the populations of the Sila and Aspromonte massifs had similar leaf traits (Table 2, Fig. 7).

## Discussion

According to the multispecies coalescent model based on ptDNA and nrDNA data, *Soldanella* underwent an evolutionary radiation during the early Pleistocene, approximately 1.78 My ago. The genus, however, came from an older lineage that diverged from *Hottonia* around 20 My ago, during the early Miocene. Our phylogeny finely matches the one derived by Yesson and co-workers [12], providing similar stem ages for *Androsace*, *Omphalogramma*, *Hottonia* and *Soldanella*. Considering the agreement between the phylogenies, it is reasonable to have a high confidence on the age estimate for the evolutionary radiation of the *Soldanella* genus. This estimate is based on the latest common ancestor of both *S. pusilla* and the metapopulation of the Gelbison massif which, as highlighted by the ITS phylogeny, belong to lineages diverged during the early differentiation of the genus, ensuring an unbiased dating of the diversification of *Soldanella*. Unlike our results, this event was previously dated [5] to the middle-late Pleistocene based on an ITS phylogeny calibrated using a fossil of *Androsace*. The fossil, however, had a broad age estimate (23.3–5.2 My) and was later discarded for calibrating the *Androsace* phylogeny by Schneeweiss and Schönswetter [13]. This, coupled with the different data and models employed, could possibly explain the discrepancies between our dating and the earlier one. However, since our estimate for the diversification of *Soldanella* is based on a model of gene coalescence within a species tree, with sequences from both plastid and nuclear DNA, and uses a robust calibration node [12], it is likely more accurate than the one previously proposed.

The phylogeographical scenario obtained from the ITS data suggests that the diversification of *Soldanella* began in middle Europe, although a Carpathians origin could not be ruled out. Assuming a central-southeastern Asian origin of the lineage, as indicated by the distribution of its closest relatives, *Hottonia* and *Omphalogramma*, the ancestor of *Soldanella* arrived in Europe during the time span of about 18 My from the divergence of the lineage to the diversification of the genus. The migration was likely triggered by the drop in Earth surface temperature since the Middle Miocene Transition (14.0–13.5 My) onward [14], as well as by the change in vegetation from thermophilous evergreen broad-leaved forests to deciduous broad-leaved and then to mixed coniferous/deciduous broad-leaved forests during the early Miocene-Pliocene [15]. Indeed, the last two vegetation types are those to which most of the species of *Soldanella* associate with [2], especially those with a montane ecology, which are considered, from an evolutionary point of view, the basalmost ones in the genus [5, 8]. The changes in vegetation could have thus created a preferential route for the spreading of the *Soldanella* lineage in Europe, where it found suitable environmental conditions for its persistence in the late Miocene-Pliocene.

According to our phylogeographical simulation, *Soldanella* extended from central Europe over the Carpathians during the early Pleistocene, and then westward to the Pyrenees and southern Italy during the middle Pleistocene. A thermal driven migration is the most probable explanation for the origin of the southern Apennine populations of *Soldanella*. Indeeed, the crossing of the Adriatic barrier thanks to the drop in sea level during cold ages is one of the most common ways for the species of the Carpathians and Balkans to reach the Italian Peninsula. Considering the low genetic variability of the populations of *Soldanella* in southern Italy, their origin can be alternatively the result of a single dispersal event, although the pattern of genetic diversity could have been altered by later population bottlenecks. Similar migration patterns were hypothesised by Surina and coworkers [16] for the Apennine populations of *Edraianthus graminifolius* (Campanulaceae).

According to the multispecies coalescent model, the diversification of *Soldanella* in southern Italy began with the differentiation of the metapopulation of the Gelbison massif as an independent evolutionary unit. The event is dated during the middle-late Pleistocene, with an indication of approximately 0.38 My ago. During this time, the 100 ky Milankovitch cycles determined repeated glaciations and interglaciations, accompanied by expansions and contractions of the species distribution ranges, which caused population bottlenecks [17–19]. Particularly in southern Italy, Balkans and Iberian peninsula, the species associated to colder climates survived the interglacials by colonising higher altitudes [20, 21], experiencing repeated habitat fragmentation. Considering the geographical isolation among the Gelbison, the Sila and the Aspromonte massifs, it is reasonable to hypothesise a vicariance process for the divergence of the populations of *Soldanella* in the southern Apennines, which could have been isolated due to the fragmentation of a larger original distribution range. Alternatively, the diversification could be the result of a long distance dispersal from the populations on the Gelbison massif to the Calabrian Apennines. However, the vicariance hypothesis seems more appropriate than this scenario, since all of the metapopulations show similar low genetic diversity, suggesting common processes for their origin. Among the studied genera, a similar vicariance hypothesis has been proposed also by Zhang and co-workers [5] for some species of *Soldanella*, by Kropf and co-workers [22] for *S. alpina* within the Central Massif in France, and by Dixon and co-workers [23] and Schneeweiss and co-workers [24] for some *Androsace* species. Moreover, a sympatric speciation due to polyploidisation/hybridization can be safely excluded since both the Sila [25] and the Aspromonte [2] populations have the same karyotype and because of the absence of other *Soldanella* lineages in southern Italy.

The different ecology and biometrical traits between the populations of the Gelbison massif and those of the Sila and Aspromonte massifs further support their assignments to two independent evolutionary units. The presence of endemic ITS haplotypes, however, suggests that the populations from the two distribution ranges on the Calabrian Apennines could be also differentiated. Such an hypothesis is supported also by the morphometric differences pertaining to their glandular hairs. Conversely, the ecological data do not offer additional support to this idea, because of the wide niche overlap between the metapopulations of the Calabrian Apennines. However, a similar ecological niche does not constitute a strong prove to consider the Sila and Aspromonte populations as a single evolutionary unit. Further analyses on the gene flow among these metapopulations and the use of different markers are required to clarify this point. On the contrary, the ecological data clearly demonstrate that the metapopulation of *Soldanella* on the Gelbison massif represents a widely differentiated entity from those of the Calabrian Apennines. The altitudinal range and the composition of the forest canopy to which the populations of *Soldanella* of the Gelbison massif are associated, deviates strikingly from those observed for the populations of the Calabrian Apennines. Considering the altitudinal range, in particular, not only does it not overlap with that of the populations of the Calabrian Apennines, but it is also unique within the entire *Soldanella* genus, whose species are commonly found at higher altitudes. Indeed, only *S. villosa* can be found at lower altitudes, but it occupies a smaller altitudinal range than the populations of the Gelbison massif which, moreover, are associated to a wider range of canopies [2, 5]. The association of the populations of the Gelbison massif to *A. cordata*, *A. glutinosa* and *C. sativa* is also unique among the *Soldanella* species, which are generally found in grassland, snow pockets, Alpine pastures, or under conifer, *F. sylvatica*, *Acer* spp. and *Sorbus* spp. canopies [5]. In this context, it is remarkable that the populations of the Sila and Aspromonte massifs are often found under mixed *A. alba* and *F. sylvatica* canopies, whereas the populations of the Gelbison massif are never found under conifers, although plantations of *Pseudotzuga menziesii* Mirb. Franco and *A. alba*, with rare *Picea abies* L. plants, are occasionally found within their distribution range on the massif. The higher pH and lower C and organic matter of the soil on which plants grow on the Gelbison massif could possibly explain this occurrence, since conifer litter usually enhances soil acidification and it accumulates due to slow decomposition rates [26]. The hydrological characteristics of the wetlands colonised by the populations of the Gelbison massif point to a higher dependency on water availability compared to those of the Sila and Aspromonte massifs, since plants colonise the brook beds and their sides nearby water. The specialization towards the brook environment of the *Soldanella* of the southern Apennines is unique within the genus [5] and is probably related to the Mediterranean-type climate of the area. The populations of the Gelbison massif, however, stand out in this context for their strict hydrological requirements, probably related to the low elevation at which plants grow, where water of the perennial brooks could mitigate summer drought constrains and decouple the plant survival from the soil water resource.

The differences in the ecology of the three metapopulations are mirrored by the quantitative differences in their morphology. The relative size of the terminal cell of leaf petioles glandular hairs, in particular, is the best criterion to differentiate the populations of the three provenances. However, a wide array of biometrical traits, related to both the glandular hair and leaf morphology, further supports the differentiation of the three metapopulations of *Soldanella* in the southern Apennines.

Finally, the *rbcL* and *trnL* sequences can be used to unequivocally differentiate the populations of the Gelbison massif from those of the Calabrian Apennines in a taxonomic context. Both of these markers are potential candidates for the universal DNA barcoding of plants [27–32], but usually possess an insufficient discriminatory power among different species. For this reason, they are commonly used in combination with other markers with a higher evolutionary rate, like the *matK*, to identify different species [29, 32]. The observed differences in the *rbcL* and *trnL* sequences between the populations on the Gelbison massif and those on the Sila and Aspromonte massifs can be thus interpreted not only in a phylogenetic, but also in a taxonomic context.

**Fig. 8:**
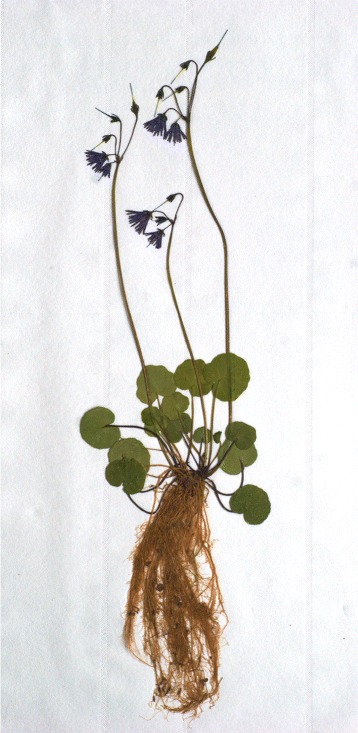
Soldanella sacra A. & L. Bellino. Holotype of *Soldanella sacra* A. & L. Bellino, *species nova*

The unique molecular, morphometric and ecological traits of the populations of *Soldanella* of the Gelbison massif are sufficient to redefine *S. calabrella* as endemic to the Calabrian Apennines, and to consider the populations of the Gelbison massif as belonging to a new taxonomic unit at the species level. We name this unit *Soldanella sacra* A. & L. Bellino (Fig. 8), from the name of the Gelbison massif, the “Holy Mountain”, *locus typicus* of the new species.

## Soldanella sacra A. & L. Bellino, species nova

### Diagnosis

Planta perennis, mediocris vel maiuscula, 15–35 cm alta. Rizomates ramosa, repentes vel ascendentes, saepe cum stolonibus. Petioli purpurei, 2.8–12.2 cm longi, non in lamina protracti. Pili in petiolis iuvenilibus unicellulares vel saepe bicellulares, globosi. Pili in petiolis vetustioribus tricellulares, 85.4–125.8 *μ*m longi. Distalis cellula eorum pyriformis, 34.2–48.1 *μ*m longa et 32.2–37.6 *μ*m lata; cellulae stipitis aequilatae et aequilongae. Laminae foliarum subcoriaceae, glabrae, reniformes et crenatae, cum manifestis venis in superiore parte, sinu basali angusto. Laminae 2.0–2.6 × 3.0–4.2 cm latae. Scapi cum 3–9 floribus, erecti. Pili in pediculi flororum tricellulares, 65.6–72.1 *μ*m longi. Distalis cellula eorum globosa. Corolla violacea vel alba, antherae violaceae vel fulvae, sagittatae. Antherarum connectivum 1.0–1.5 mm longum, stylus exsertus.

### Holotype

Gelbison massif, specimen with three flowering scapes grown at 1100 m a.s.l on a brook bank under *C. sativa* canopy, 40.21225 °N - 15.31623 °E, collected on the 28^th^ of April 2010 and stored at the “Erbario Centrale Italiano” of Florence (Italy) since the 16^th^ of June 2010 (Fig. 8).

### Isotype

Gelbison massif, specimen with a single flowering scape grown at 1100 m a.s.l on the downward side of a small rock island in a brook bed under *C. sativa* canopy, 40.21225 °N - 15.31623 °E, collected on the 28^th^ of April 2010 and stored at the “Erbario Centrale Italiano” of Florence (Italy) since the 16^th^ of June 2010.

### Description

Small plant, 15–35 cm tall. Rhizomes branched, creeping or ascending, sometimes with stolons, with primary roots ∼1 mm in diameter at the base. Leaves (Additional file 2: Figure S1) with green lamina, deeply cordate at the base, 1.4–4.5 cm long and 1.8–5.0 cm wide, adaxial side with marked veins, unclear abaxially. Petioles 2.8–12.2 cm long, red, violet or sometimes green, with two distinct types of glandular hairs, both stalked and non-stalked (or shortly stalked). Young petioles and leaf primordia usually densely covered only by non-stalked glandular hairs, old petioles covered by both types. Non-stalked glandular hairs (Additional file 2: Figure S2) of one spherical cell, 32.8–40.0 *μ*m in diameter, brownish-yellow, often shortly stalked on a small cell ∼13 *μ*m tall. Stalked glandular hairs (Additional file 2: Figure S2) straight, 85.4–125.8 *μ*m long and constituted by 3 cells (exceptionally by 4 cells), with the terminal cell pyriform, 34.2–48.1 *μ*m long, with maximum diameter of 32.2–37.6 *μ*m and minimum of 17.1–20.9 *μ*m (see also Table 2). Terminal cell red or rarely non-coloured, containing numerous small vesicles. Scapes one or many per rhizome, 11.5–23.1 cm long, light to dark green, sparsely covered with non stalked glandular hairs similar to those on young petioles. Bracts in the same number of pedicels, dark green, 3.0–5.0 mm long and 1.0–1.4 mm wide at the base, with no marked veins and with few non-stalked glandular hairs. Pedicels, 3–7 (max 9) per plant, reclined during flowering, then erect, 1.0–2.5 cm long at flowering, 3.2–4.8 cm at fruiting, densely covered with stalked glandular hairs toward the calyx. Glandular hairs on pedicels (Additional file 2: Figure S2) stalked, straight, 65.6–72.1 *μ*m long, constantly of 3 cells. Cells of the stalk 18.0–23.2 *μ*m long, basal cell clearly conical, middle cell barrelled or conical; stalk in the complex markedly conical, of the same color of the terminal cell. Terminal cell almost spherical, 28.3–32.2 *μ*m long and 30.9–34.8 *μ*m wide, red, with many small vesicles. Calyx lobes (Additional file 2: Figure S3) 3.0–5.0 mm long and 1.0–1.2 mm wide at the base, with no marked veins. Calyx lobes almost glabrous on the abaxial side, with many non-stalked or shortly stalked glandular hairs on the adaxial side, 36.7–39.4 *μ*m wide in diameter. Corolla violet, occasionally white (Additional file 2: Figure S3), regularly to irregularly incised. Corolla tube 3.5–5.7 mm long, shorter or rarely equal to the lobes, 6.2–9.6 mm long. Stamens and connectives without glandular hairs or occasionally with stalked glandular hairs of 2 cells. Connectives white, 1.1–1.7 mm long, shorter than filaments, slightly to deeply lobate at the apex. Filaments purple, 1.8–2.6 mm long. Anthers yellow or violet, acute at the base, 2.1–3.0 mm long, with connective appendages usually darker than anthers, 1.0–1.7 mm long. Pollen grains (Additional file 2: Figure S4) elliptical and trisyncolpate during pollination (dried), on average 20.3 ×11.8 *μ*m, the same spherical when hydrated, on average 20.3 *μ*m in diameter, with a smooth surface. Style 8.5–10.5 mm long, longer or equal to the corolla. Capsules (Additional file 2: Figure S5) 11.7–14.8 mm long at maturity, with 10–11 min teeths. All measures are given as the range between the 5^th^ and the 95^th^ quantile.

### Etymology

The epithet *sacra* refers to the massif on which this species was discovered, named the “Holy Mountain” or Gelbison, likely from the ancient Arabic “Gebil el son” which stands for “The mount of the Idol”. Historically the massif was an important religious site in the Mediterranean Basin, as also testified by the presence of an ancient Heraion on the peak, replaced since 1323 A.D. by a sanctuary dedicated to the Marian cult.

### Distribution and ecology

At the present, all the populations of *S. sacra* are found on the Gelbison massif, in the Campanian Apennines.

The typical micro-environment of this species is characterized by small islands of soil or stones scattered in the brook bed and under small cascades, usually colonized on the downward side and covered by mosses and liverworts (Additional file 2: Figure S6). *S. sacra* associates primarily and almost constantly with the mosses and liverworts that cover the substrate, mainly constituted by *Bryopsida* and *Marchantiales*. It can also be found associated with many other species, either typical of the same environment, like *Lysimachia nemorum* L., *Juncus effusus* L. and *Nasturtium officinale* R., or less specialised like *Chaerophyllum hirsutum* L., *Anthriscus nemorosa* L., *Lamiastrum galeobdolon* L., *Mycelis muralis* L., *Adenostyles australis* Ten., *Geranium robertianum* L., *Geranium versicolor* L., *Festuca heterophylla* Lam., *Oxalis acetosella* L., *Bellis sylvestris* Cyr., *Saxifraga rotundifolia* L., *Cystopteris fragilis* L. and *Polystichum aculeatum* L. In relation to the tree canopy, *S. sacra* is strictly associated to *A. cordata* and *A. glutinosa*, which are sometimes associated with man-made *C. sativa* stands and occasionally with *Ilex aquifolium* L., *Corylus avellana* L., *Carpinus betulus* L. and *Fraxinus ornus* L. Approximating the higher altitudinal limit, *S. sacra* can be found also in Mediterranean microthermal *F. sylvatica* forest type. The species is found in an altitudinal range of 840 - 1400 m a.s.l., on neutral soils with an organic matter content on average equal to 15 % d.w. and a C/N ratio of ∼12. *S. sacra* flowers from April to May and fructifies from June to July, dispersing seeds mainly through hydrochory.

### Conservation strategies

To date, *S. sacra* is represented by only ten spatially separated populations on the Gelbison massif (Additional file 2: Figure S7). Therefore, this new species could be considered as a narrow stenochorous endemism of the mountain forest ecosystems of the southern Apennines. As far as we know, *S. sacra* is the species with the most narrow distribution range within its genus. The extent of the area occupied on the Gelbison massif, which is limited to very narrow patches, could have been restricted during the last decades by the plantations of the alien conifers *P. menziesii* and *P. abies* on the lower altitudinal range where *S. sacra* finds its habitat. These plantations modified the edaphic conditions and the light regime under the canopy, which could have determined the local extinction of the species. Today, the occurrence of *S. sacra* is limited to some of the few brook segments still flowing under *A. cordata*, *A. glutinosa* and *C. sativa* canopies. These sub-montane forests are partially of anthropogenic origin and testify a modification of the soil use during the last 60 years: *C. sativa* woods replaced the native mesic forests and *A. cordata* recolonised the old pastures and cultivations, while *A. glutinosa* has always been present along the brook sides. The exclusion of cow grazing (cows occasionally eat *S. sacra* plants and destroy them with their hooves) and anthropogenic interferences (like the brook regimentations and the banks disruption by the passage of off-road vehicles), together with the maintenance of the typical canopy cover, are crucial to conserve the main population stock of *S. sacra*. The brook regulation, however, can also potentially promote the expansion of the populations of *S. sacra*, by managing the water flow and creating suitable habitats. An example in case is the G2 population (Table 1, Additional file 2: Figure S7), that colonised a man-made dike on a steep slope which created a suitable habitat for *S. sacra*. Since the population found at the highest elevation grow under the canopy of an old coppice beech stand, the distribution of *S. sacra* seems to be affected also by the light regime, as also demonstrated for *S. hungarica* [33]. Forest management strategies should thus promote “light windows” by opening the beech canopy located in the higher brook sections, with and without *S. sacra* populations. This would favour the propagation and/or translocation of the plants where they are not present today. A final remark should be made about the metapopulation dynamics. The environments colonised by *S. sacra*, indeed, are spatially and temporally unstable, as testified by a mudslide that occurred in the area of the G5 population and a rockslide that occurred in the area of the G7 population during the three-years monitoring performed in the present study. Such events determined heavy losses that could even determine local extinction. Since the numerical bottlenecks to which the population can undergo are potentially dangerous for the long term viability of *S. sacra*, the understanding of the metapopulation dynamics is a pivotal step in defining suitable conservation strategies. According to the IUCN Red List Categories and Criteria version 3.1 [34], *S. sacra* is to be considered as critically endangered, because of the area of occurrence smaller than 100 km^2^, coupled with a highly fragmented area of occupancy, and extreme fluctuations in the number of individuals, in the area of occupancy and in the number of subpopulations. In addition, the agamic reproduction via stolons is very common in this species, thus reducing the genetic variability. However, it must be considered that large sectors of the Campanian Apennines, where this species was found, are scarcely explored [35] and thus it is possible that the actual distribution range of *S. sacra* could be more extensive than it is known to be at present. Fortunately, the Gelbison massif and the neighbouring mountains are already included in the Cilento, Vallo di Diano and Alburni National Park, which could promote appropriate conservation strategies.

## Methods

### Study areas

Fifteen populations of *Soldanella* sp. were studied during the years 2010–2013 on the southern Apennines (Fig. 1). Specifically, eight populations were studied on the Gelbison massif (1705 m a.s.l.), five on the Sila massif (1928 m a.s.l.) and two on the Aspromonte massif (1956 m a.s.l.). The three massifs have different ontogeny and geological characteristics. The Sila [36] and Aspromonte [37] massifs developed earlier than the Gelbison massif and, being part of the Calabrian-Peloritan Arc, have mostly a granitic crystalline structure, whereas the Gelbison massif has a complex structure of different layers of permeable conglomerates and sand-stones that lay on an impermeable marly-clay olistostrome [10, 38].

A Colorado 300 (Garmin International, Kansas City, USA) GPS was used to georeference the studied populations, of which positions, altitudes and tree canopy compositions are reported in Table 1.

### Ecology

To characterise the soil of the studied populations, three samples in the layer 0–5 cm were collected from the rhizosphere of each sampled plant and pooled together. Samples were oven dried at 75 °C and sieved to 2 mm to retrieve the granulometric fraction. The following parameters were measured on each sample: i) pH in distilled water (1:5 w:w soil:water) by a FiveGo pH-meter (Mettler-Toledo, Schwarzenbach, Switzerland), ii) total C and N through a Flash EA 1112 CHNS-O Analyzer (Thermo Fisher Scientific Inc., Waltham, MA, USA) after pulverisation with a PM 4 planetary ball mill (Retsch, Haan, Germany), iii) soil organic matter (OM) via loss on ignition with a Controller B 170 furnace (Nabertherm GmbH, Lilienthal, Germany) for 4 h at 550 °C.

To test the differentiation of the populations from the three provenances based on soil rhizosphere characteristics, tree canopy composition and site elevation, Non-metric Multidimensional Scaling (NMDS) with the superimposition of the confidence ellipses (for *α* = 0.05) for the three metapopulations was performed. In order to accommodate the presence of mixed data in the data matrix, the Gower distance metric was used. In addition, one-way Analysis of Variance (ANOVA), followed by the Tukey HSD *post hoc* test, was used to test the differences among the three metapopulations for each of the soil characteristics analysed. The analyses were performed using R programming language with the “vegan” package [39] and the “stats” package [40].

### Molecular analyses

One nuclear (total ITS) and two plastid (*trnL* and *rbcL*) molecular markers were analysed for the phylogenetic analysis of the 15 studied populations. Three plants were sampled for the analyses from the edges of each population (six for the populations of the Aspromonte massif), in order to avoid the collection of clones. Total ITS and *rbcL* were amplified and sequenced for all the samples, whereas *trnL* was analysed on 21 samples: 6 from the Gelbison, 12 from the Sila and 3 from the Aspromonte massifs.

Total DNA was extracted using the REDExtract-N-Amp™ Plant PCR kit (Sigma-Aldrich Corp., MO, USA) from ∼ 2 mm^2^ of a healthy, young leaf per sample. Amplification was carried out by Polymerase Chain Reaction (PCR), using the “trnL 1” (5’-CGAAATCGGTAGACGCTACG-3’) / “trnL 2” (5’-GGGGATAGAGGGACTTGAAC-3’), “rbcL 1F” (5’-ATGTCACCACAAACAGAAACTAAA-3’) / “rbcL 1375R” (5’-AATTTGATCTCCTTCCATATTTCGCA-3’) and “ITS 1a” (5’-TCGTAACAAGGTTTCCGTAGG-3’) / “ITS 28kj” (5’-CTTGGACGGAATTTACCG-3’) primer pairs for *trnL*, *rbcL* and total ITS (ITS1 + 5.8S + ITS2), respectively. The amplifications of *trnL* and *rbcL* were performed in a volume of 20 *μ*L, containing 10 *μ*L of RedTaq™ polymerase, 2 *μ*L of each primer (at a final concentration of 0.5 *μ*M), 2 *μ*L of DNA extract and 4 *μ*L of distilled and sterile water. The amplification of total ITS, instead, was performed in a volume of 10 *μ*L, with halved volumes of each constituent.

Double stranded DNA templates were then produced in a 2720 thermal cycler (Applied Biosystems, Foster City, CA, USA), using different protocols. For *trnL* and total ITS, an initial step of 3’ at 94 °C was followed by 32 cycles, each consisting of denaturation for 30" at 94 °C, annealing for 1’ at 55 °C and elongation for 9’ at 72 °C. For *rbcL*, the initial step of 3’ at 94 °C was followed by 35 cycles, each consisting of denaturation for 30" at 94 °C plus 3”/cycle, annealing for 1’ at 50 °C and elongation for 9’ at 72 °C. Amplicons were purified using the Illustra GFX PCR DNA and GCI Bind purification kit™ (GE Healthcare Bio-Sciences Corp., Piscataway, NJ, USA).

Purified DNA was amplified using BigDye Terminator Cycle Sequencing Kit (Applied Biosciences, Connecticut, USA), using the same protocols and primers of the initial PCRs but in different forward and reverse reactions. Sequencing PCR were performed for each marker in a 5 *μ*L volume, containing 1 *μ*L of BigDye™, 1 *μ*L of reaction buffer, 0.5 *μ*L of the primer (at a final concentration of 0.16 *μ*M) and water if needed. DNA was used at a final concentration of 6 ng/ *μ*L for *trnL*, 14 ng/ *μ*L for *rbcL* and 10 ng/ *μ*L for total ITS. Sequences were analysed using an ABI Prism 310 automatic sequencer (Applied Biosystems, Foster City, CA, USA).

Sequences of each unique haplotype or ribotype were deposited in GenBank (accessions KT368181-KT368190).

The 222 electropherograms (forward and reverse) obtained were analysed using the Chromas Lite software (Technelysium Pty Ltd, Brisbane, Australia). The sequences were then built and edited using a text editor, and aligned using the ClustalX software [41]. To build the phylogenies and calibrate the trees, sequences of other Primulaceae and Myrsinaceae were retrieved from GenBank using the BLAST software. Sequences of *Androsace septentrionalis* (JX848513.1 and AF394963.1 for *rbcL*, EU326038.1, AY274959.1, EU655581.1 and GQ244557.1-8.1 for *trnL*, AY275074.1 and EU655561.1 for ITS), *Cyclamen hederifolium* (U96656.1 for *rbcL*, AM990526.1 and AJ236994.1-5.1 for *trnL*, AF164004.1, AJ491440.1 and AJ491684.1 for ITS), *Omphalogramma delavayi* (AF213805.1 and JF942659.1-63.1 for *rbcL*, DQ378603.1 and AF534675.1 for *trnL*, JF977173.2-7.2 for ITS), *Hottonia palustris* (AF395002.1 and KM360824.1 for *rbcL*, AF402435.1 for *trnL*, AJ491430.1 and AJ491674.1 for ITS1 and ITS2) and *S. pusilla* (AF395000.1 for *rbcL*, AF402434.1 for *trnL*, AJ306353.1-8.1 for ITS) were chosen, in relation to their similarities with *S. calabrella* and the availability of all the markers for each species. These sequences were employed in dating the diversification of the *Soldanella* genus and of the metapopulations of the southern Apennines based on all the markers analysed using Bayesian techniques. For the phylogeny of the entire *Soldanella* genus, based on the ITS marker, the sequences of all the species available in GenBank with an indication of the provenance (accessions AJ306321.1-361.1 and KJ365400.1-17.1), obtained by Zhang and co-workers [5] and by Steffen and Kadereit [8], were analysed employing both maximum likelihood and Bayesian techniques.

The Bayesian analyses were performed using BEAST 2.3.0 [42] and Tracer 1.6 to analyse and combine the MCMC chains. To estimate both phylogeny and divergence times, the *rbcL*, *trnL* and ITS alignments were analysed jointly with a multispecies coalescent model (*BEAST), which takes into account the species divergence processes from multilocus DNA data. For the *rbcL* and ITS alignments, a General Time Reversible substitution model (with the estimation of the equilibrium frequencies, the proportion of invariant sites, and the *Γ* distribution with 4 discrete classes) and a calibrated Yule tree model were employed, together with a relaxed clock lognormal model. For the *trnL* alignment a strict clock model was chosen instead, based on the evaluation of the coefficient of variation of a relaxed clock lognormal model, as well as the same site and tree model of the other markers. Since the plastid markers belong to a single non-recombinant DNA molecule, a single tree was estimated for the ptDNA data. To this end, the missing part of *rbcL* or *trnL* gene for each sequence was coded as missing data, so the effective number of sequences for the ptDNA analysis is the sum of the sequences for each of the plastid markers. The analysis was performed combining the results of 5 runs with 3.5∗10^8^ generations, sampled every 5∗10^3^ iterations. The phylogeny was calibrated using the divergence between Primulaceae and Myrsinaceae, dated to 44.6–45.3 My ago by Yesson and co-workers [12] and constraining the monophyly of the Primulaceae to improve convergence of the MCMC chains.

The maximum likelihood estimation of the ITS phylogeny was performed using the PhyML 3.0 software [43], starting from a BioNJ tree based on Kimura-Nei distances [44]. All the available substitution models in PhyML were tested for each marker using the “phymltest” function of the “ape” package [45] in R. The General Time Reversible [46] model, with the estimation of the equilibrium frequencies, the proportion of invariant sites, and the *Γ* distribution (with 4 discrete classes), was thus chosen for all the alignments in relation to its lowest Akaike Information Criterium. Tree topologies were optimized using the best of NNI and SPR moves, and branch support was based on 1∗10^3^ bootstrap replicates.

The Bayesian analysis of the ITS alignment involved the construction of a discrete phylogeographical scenario for the evolution of the *Soldanella* genus. A General Time Reversible model with the estimation of the equilibrium frequencies, the proportion of invariant sites and the *Γ* distribution (with 4 classes) was employed for the substitution model of the ITS alignment, whereas a symmetric model was chosen for the matrix of discrete traits containing the provenances of the *Soldanella* sequences. The provenances were divided into 6 discrete regions: 1) Carpathians, 2) central Europe, 3) Balkans, 4) Alps, northern, and middle Apennines, 5) Pyrenees, and 6) southern Apennines. A calibrated Yule tree model and a strict clock model, chosen by examining the distribution of the coefficient of variation of a previous relaxed clock lognormal model, were employed. The calibration of the ITS tree was based on the dating of the *Soldanella* radiation obtained from the *BEAST analysis. The analysis was performed combining the results of 5 independent runs each with 5∗10^7^ generations, sampled every 5∗10^3^ iterations. Finally, the phylogeny was combined with the geographical information on the sequence provenances using the SPREAD 1.0.6 software [47].

### Morphometry

Biometrical measurements were performed on 10–20 fresh plants collected from each population. Observations and measurements of the glandular hairs were carried out with a Leitz Dialux 20 microscope at 100-400x magnification in bright field and phase contrast, equipped either with a Photometrics CoolSnap K4 camera (Roper Scientific, AZ, USA) or a Nikon DS-U2 digital camera (Nikon Corporation, Tokyo, Japan). For each glandular hair of leaf petioles, the length (l), minimum width (wmin) and maximum width (wmax) of the three cells (terminal: t; middle: m; basal: b) were measured, as well as the total hair length (h). In the rare cases of glandular hairs with four instead of three cells, the measurements were carried out on the terminal cell, the basal cell and the cell adjacent to the basal one (m). Moreover, the maximum length (le), the length from the petiole to the apex (lc) and the maximum diameter (d) of each leaf as well as the length of leaf petioles (lp) were also measured with the ImageJ 1.43u software package on images collected with an Epson Perfection 1640 scanner (Seiko Epson Corporation, Nagano, Japan) at a resolution of 1600 dpi. For the populations of the Gelbison massif only, in addition, plant and scape heights were measured with a manual vernier caliper, floral traits were measured *via* image analysis after longitudinal sectioning the flowers to expose internal structures, and pollen grains were observed with the same microscopy apparatus in bright field at 1000 × magnification.

The differentiation of the populations from the three provenances based on glandular hair and leaf biometrical traits was evaluated with a Canonical Variates Analysis (CVA), performed using the “candisc” 0.6–5 package [48], and with one-way ANOVAs followed by Tukey HSD *post hoc* tests, as described for the ecological data.

## Additional files

Additional file 1
**Video S1.** Phylogeographical scenario for the diversification of the *Soldanella* genus during the Pleistocene. (OGV 3153 kb)

Additional file 2
**Figure S1.** Abaxial (left) and adaxial (right) side of the leaves of *Soldanella sacra*. **Figure S2.** (a) stalked glandular hair of leaf petioles, (b) stalked glandular hair of pedicels and (c) non-stalked glandular hair of the adaxial side of calyx lobes. Glandular hairs in (c) are morphologically similar to the non-stalked glandular hairs of leaf petioles. Black bar = 10 *μ* m, scale is the same for all the images. **Figure S3.** Normal (left) and white variant (right) of the corolla of *Soldanella sacra*. **Figure S4.** Dried (a, b) and hydrated (c) pollen grains of *Soldanella sacra*. (a) and (b) show the same pollen grain respectively in transversal and in longitudinal view. Black bar = 10 *μ* m, scale is the same for all the images. **Figure S5.** Capsules of *Soldanella sacra* after seed dispersion with visible minute theets. **Figure S6.** Plants of *Soldanella sacra* living in their habitat. **Figure S7.** Populations of *Soldanella sacra* on the Gelbison massif. The two populations not included in the study are highlighted in orange. (PDF 2252 kb)
