# Supplementary material for: Evolution, ecology and systematics of Soldanella (Primulaceae) in the southern Apennines (Italy)
Source: BMC Evol Biol. 2015 Aug 11;15:158. doi: 10.1186/s12862-015-0433-y (PMC4542100; doi:10.1186/s12862-015-0433-y)
Supplement: Additional file 2 — Figure S1. Abaxial (left) and adaxial (right) side of the leaves of Soldanella sacra. Figure S2. (a) stalked glandular hair of leaf petioles, (b) stalked glandular hair of pedicels and (c) non-stalked glandular hair of the adaxial side of calyx lobes. Glandular hairs in (c) are morphologically similar to the non-stalked glandular hairs of leaf petioles. Black bar = 10 μ m, scale is the same for all the images. Figure S3. Normal (left) and white variant (right) of the corolla of Soldanella sacra. Figure S4. Dried (a, b) and hydrated (c) pollen grains of Soldanella sacra. (a) and (b) show the same pollen grain respectively in transversal and in longitudinal view. Black bar = 10 μ m, scale is the same for all the images. Figure S5. Capsules of Soldanella sacra after seed dispersion with visible minute theets. Figure S6. Plants of Soldanella sacra living in their habitat. Figure S7. Populations of Soldanella sacra on the Gelbison massif. The two populations not included in the study are highlighted in orange. (PDF 2252 kb) [file 12862_2015_433_MOESM2_ESM.pdf]

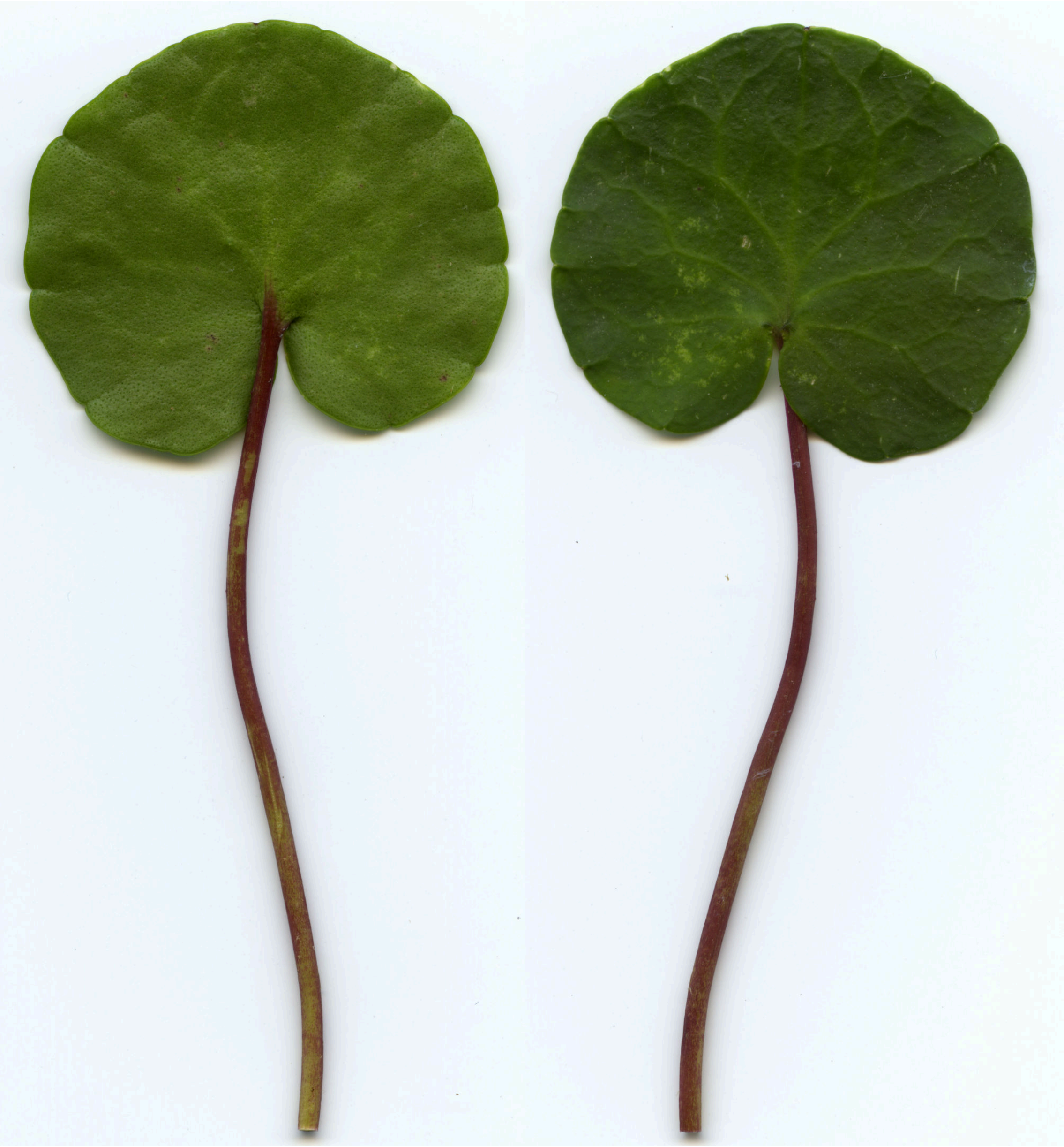

Figure S1 Abaxial (left) and adaxial (right) side of the leaves of *Soldanella sacra.*

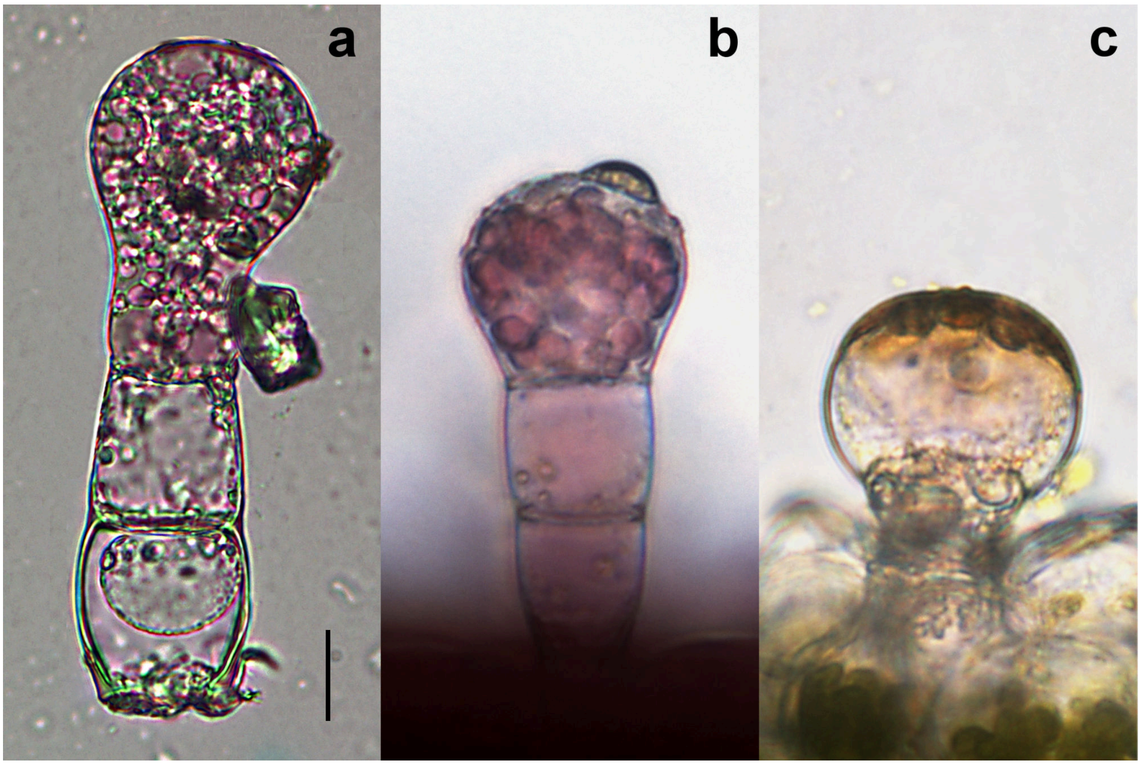

Figure S2 (a) stalked glandular hair of leaf petioles, (b) stalked glandular hair of pedicels and (c) non-stalked glandular hair of the adaxial side of calyx lobes. Glandular hairs in (c) are morphologically similar to the non-stalked glandular hairs of leaf petioles. Black bar = 10 μm, scale is the same for all the images.

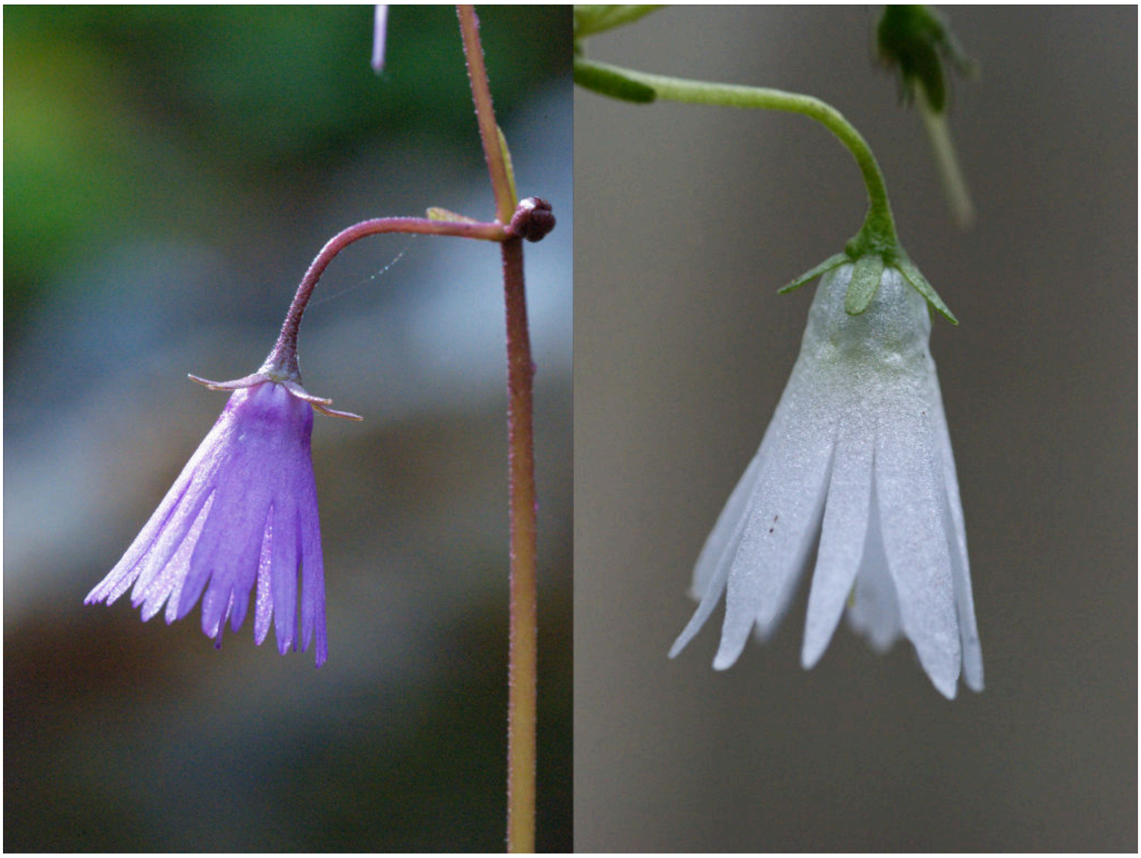

Figure S3 Normal (left) and white variant (right) of the corolla of *Soldanella sacra*.

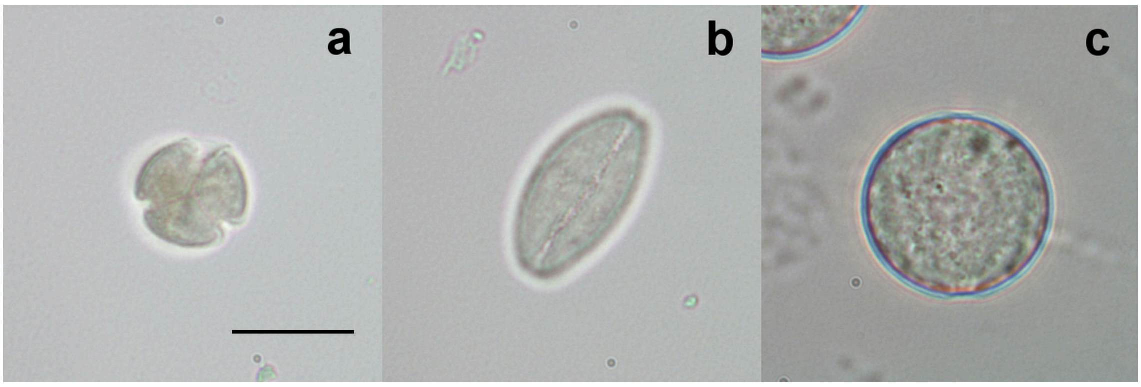

Figure S4 Dried (a, b) and hydrated (c) pollen grains of *Soldanella sacra*. (a) and (b) show the same pollen grain respectively in transversal and in longitudinal view. Black bar = 10 μm, scale is the same for all the images.

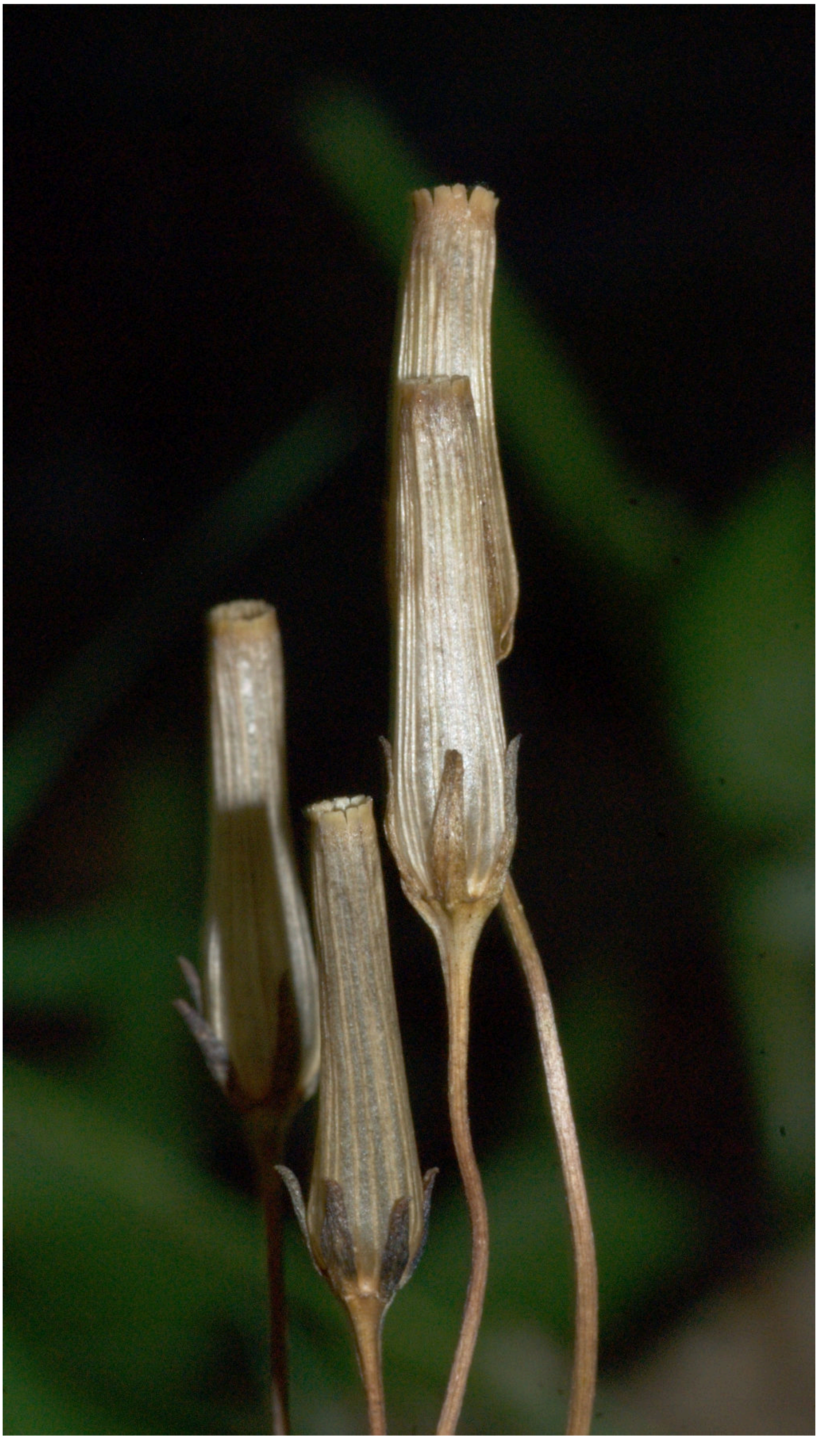

Figure S5 Capsules of *Soldanella sacra* after seed dispersion with visible subnull theets.

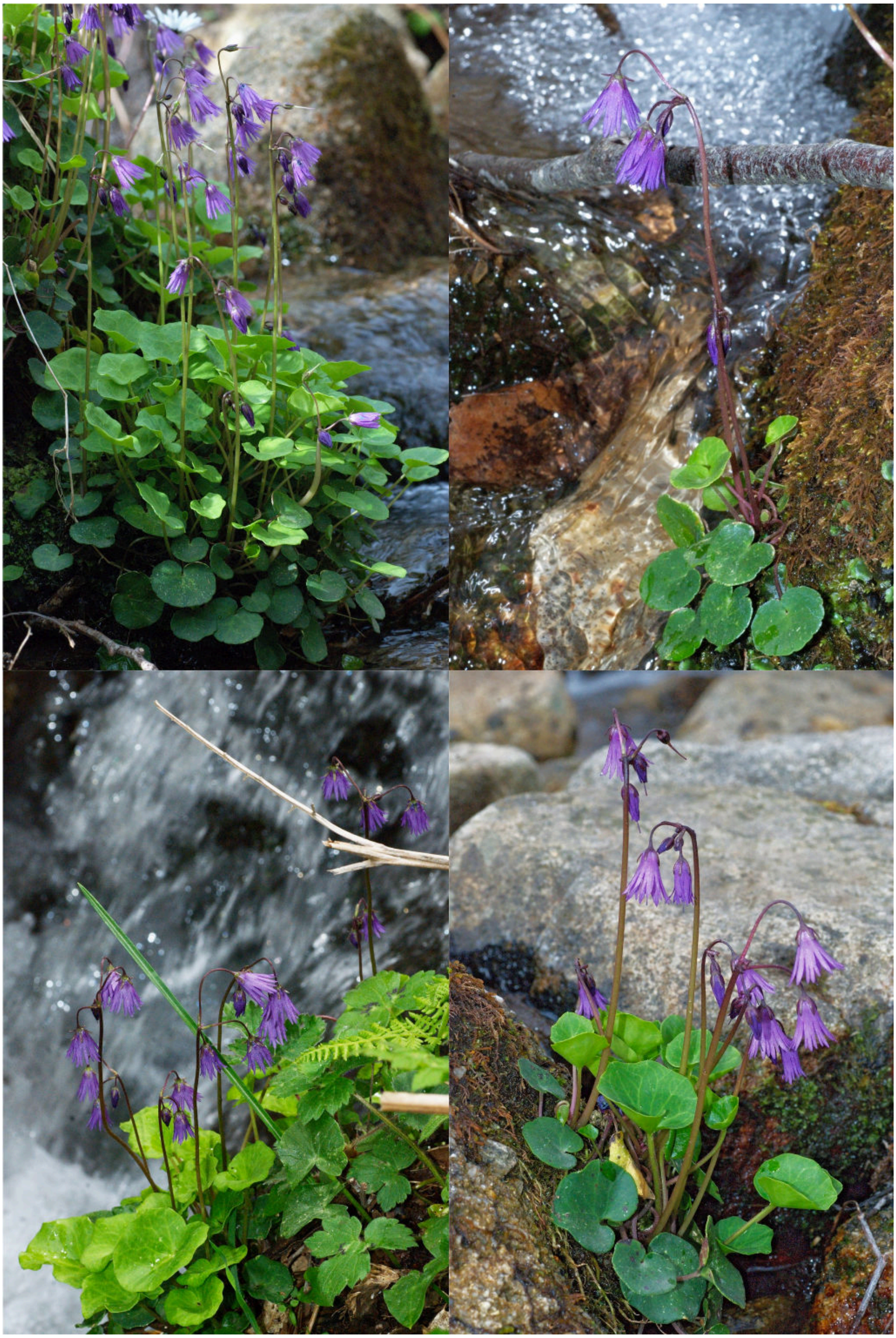

Figure S6 Plants of *Soldanella sacra* living in their habitat

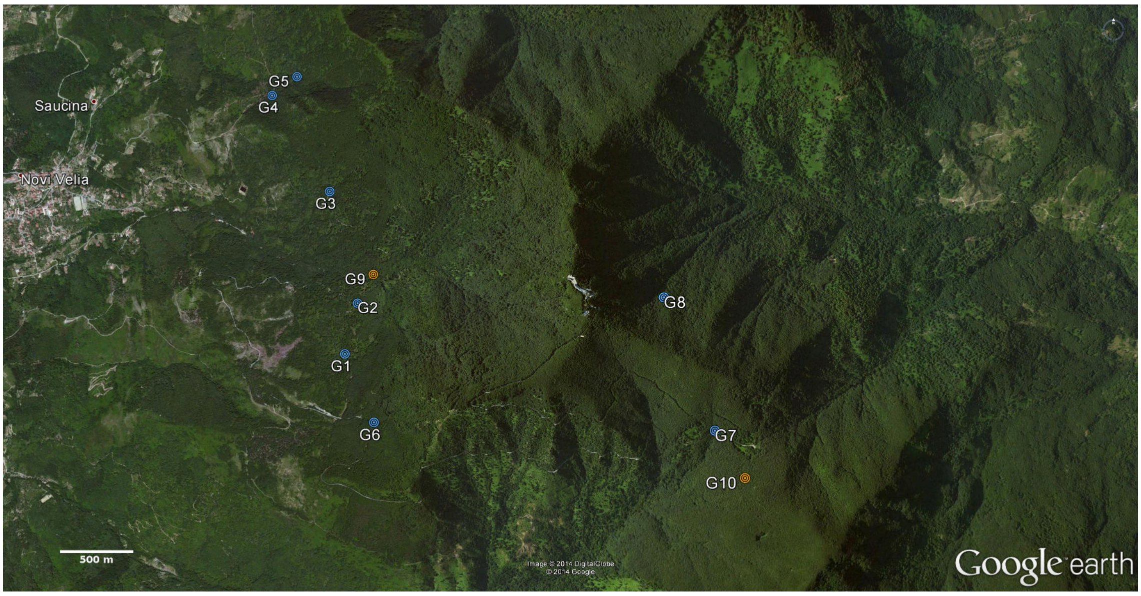

Figure S7 Populations of *Soldanella sacra* on the Gelbison massif. The two populations not included in the study are highlighted in orange.
